# Molecular markers based on sequence variation in *BoFLC1.C9* for characterizing early- and late-flowering cabbage genotypes

**DOI:** 10.1186/s12863-019-0740-1

**Published:** 2019-04-27

**Authors:** Md. Abuyusuf, Ujjal Kumar Nath, Hoy-Taek Kim, Md. Rafiqul Islam, Jong-In Park, Ill-Sup Nou

**Affiliations:** 1 Department of Horticulture, Sunchon National University, 255 Jungang-ro, Suncheon, Jeonnam 57922 Republic of Korea; 2 Department of Agronomy, Patuakhali Science and Technology University, Patuakhali, 8602 Bangladesh; 3 Department of Genetics and Plant Breeding, Bangladesh Agricultural University, Mymensingh, 2202 Bangladesh; 4 University–Industry Cooperation Foundation, Sunchon National University, 255 Jungang-ro, Suncheon, Jeonnam 57922 Republic of Korea

**Keywords:** F7R7 marker, *FLC*, Early- and late-flowering, Cabbage

## Abstract

**Background:**

Cabbage (*Brassica oleracea var. capitata*) is popular worldwide for consumption as a leafy vegetable. Premature flowering is triggered by low temperature, and deteriorates quality of cabbage as vegetable. In general, growers prefer late-flowering varieties to assure good quality compact head. Here, we report *BoFLC1.C9* as a gene with clear sequence variation between cabbage lines with different flowering times, and proposed as molecular marker to characterize early- and late-flowering cabbage lines.

**Results:**

We identified sequence variation of 67 bp insertions in intron 2, which were contributed in flowering time variation between two inbred lines through rapid down-regulation of the *BoFLC1.C9* gene in early-flowering line compared to late-flowering one upon vernalization. One set of primer ‘F7R7’ proposed as marker, of which was explained with 83 and 80% of flowering time variation in 141 F_2_ individuals and 20 commercial lines, respectively.

**Conclusions:**

This F7R7 marker could be used as genetic tools to characterize flowering time variation and to select as well to develop early- and late-flowering cabbage cultivars.

**Electronic supplementary material:**

The online version of this article (10.1186/s12863-019-0740-1) contains supplementary material, which is available to authorized users.

## Background

Cultivated as a leafy vegetable, cabbage belongs to the *Brassicaceae* family and is popular worldwide. Flowering makes rapid elongation of the stem linked to the development of an indeterminate inflorescence [1] and the crucial transition from the vegetative phase to the reproductive phase of the plant’s life cycle [2], which reduces its market quality and consumer preference. Early-flowering at premature condition leads in reduction of yield and commercial values. Therefore, vegetable growers prefer late-flowering varieties to produce high quality and economically valuable vegetables [3, 4]. Considering the value of early- and late-flowering cabbage varieties, it is important to predict flowering time before planting. The genetic features of flowering pathways, which are mediated by environmental signals, have been previously well characterized in *Arabidopsis thaliana* [5, 6].

The *FLOWERING LOCUS T* (*FT*), *SUPPRESSOR OF OVEREXPRESSION OF CONSTANS1* (*SOC1*), and *LEAFY* (*LFY*) genes are characterized through functional analysis and denoted as the main floral integrators [7–9]. *FT* acts as florigen and this protein is conserved in the most flowering plants [10]. *SOC1* and the transcription factor *LFY* encoding MADS-box protein, of which act as floral activator for controlling floral patterning and floral meristem to identity the male and female reproductive organs during the process of flower development [11–13]. In *Brassicaceae*, expression of the key gene *FLOWERING LOCUS C* (*FLC*) is regulated by the perception of vernalization (passing the vegetative phase at low temperature for certain duration) [14, 15]. MADS-box transcription factor is encoded by the *FLC* genes and repressed flowering of the plants through inhibiting downstream floral integrator genes [6, 16–20].

*Brassica* plants showed natural variation in flowering time, which provides an excellent resource for explaining the molecular mechanism behind it. In *B. oleracea*, four *BoFLC*s have been identified [21], but differences in the alleles of these *FLC* genes have not been confirmed; thus it is unknown how they were contributed in variation of flowering time? A functional allele of *BoFLC2* was identified in an annual *Brassica* [15], the mutant allele *boflc2* explained the role of *FLC* in *B. oleracea* and *Arabidopsis*. A recent study on sequence polymorphism of four *FLC* paralogs in *B. oleracea* indicated that they are not candidate in flowering time variation [22].

Many crops of *B. oleracea* species show remarkable morphological diversity and popular for their diverse edible parts, like inflorescences, axillary buds, leaves and stems [23]. Presently, two reference genomes of *B. oleracea* are available [24, 25], however based on only one reference sequence is not enough to collect the entire gene repertoire in the species, like structural variants, presence/absence variants (PAVs) and copy number variants [26–28]. A pangenome has been published for explaining such variation in *B. oleracea* [23]. Tettelin et al. [29] introduced the concept of pangenome in 2005 for full genomic makeup of a given species, which represents possible structural variation absent in the reference sequence.

To date, there has been little analysis of the molecular markers involved in the variation of flowering time by *FLC* in cabbage (a sub-family member of *B. oleracea*). Herein, we propose molecular markers based on pangenome data of flowering integrator genes of *B. oleracea* for characterization of early- and late-flowering genotypes before planting in the field.

## Results

### Selection of the genes and their phylogenetic positions

The *AtFLC* and central floral integrator genes of *Arabidopsis thaliana* [*AtFT*, *AtSOC1*, *AtLFY*, *AtCO* (*CONSTANS LIKE*), *AtVRN* (*VERNALIZATION*), *AtSVP* (*SHORT VEGETATIVE PHASE*) and *AtSPL* (*SQUAMOSA PROMOTER BINDING LIKE*)] sequences were collected from TAIR (https://www.arabidopsis.org/) and a syntenic gene search for *B. oleracea* using the BRAD database (http://brassicadb.org/brad/) retrieved syntenic genes of *BolFLC*, *BolFT*, *BolSOC1*, *BolLFY*, *BolCO*, *BolVRN*, *BolSVP*, and *BolSPL*. The genes were also cross-checked in the Bolbase (http://www.ocri-genomics.org/bolbase/genes.htm). Finally, *Hidden Markov* Models (HMM) profiling against EnsemblPlants (http://plants.ensembl.org/) and Bolpangenome (http://www.brassicagenome.net/) revealed 3, 2, 3, 1, 6, 3, 2 and, 5 *BoFLC*, *BoFT*, *BoSOC1*, *BoLFY*, *BoCO*, *BoVRN*, *BoSVP*, and *BoSPL* genes, respectively in *B. oleracea* (Table 1).

**Table 1:** In silico analysis of 25 flowering pathway genes collected from the Bolpangenome database for their protein length, chromosomal location (based on the Bolpangenome and EnsemblPlants database), and distribution of domains for each gene

Gene Name	ID	Protein (aa)	Chromosomal Location		Domains
			Bolpangenome	Ensemblplants	
*BoFLC1.C9*	Bo9g173400	197	C9:54424498–54,428,601 (+)	C9:51033935–51,038,038 (+)	MADS-box superfamily, K-box, MADS MEF2-like
*BoFLC3.C3*	Bo3g005470	197	C3:2396772–2,400,061 (−)	C3:2126639–2,129,928 (−)	MADS-box, K-box, MADS MEF2-like
*BoFLC4.C3*	Bo3g100540	200	C3:39412762–39,416,761 (−)	C3:35746750–35,750,749 (−)	MADS AFFECTING FLOWERING 5-like isoform X1, MADS-box, K-box
*BoFT.C5*	Bo5g025100	174	C5: 9412613–9,414,217 (−)	C5: 8644739–8,646,343(−)	Phosphatidylethanolamine-binding protein
*BoFT.C8*	Bo8g104520	173	C8: 39187856–39,190,040 (+)	C8: 36663547–36,665,731(+)	Phosphatidylethanolamine-binding protein
*BoSOC1.C3*	Bo3g038880	213	C3: 17200121–17,202,561 (−)	C3: 15404837–15,407,277(−)	MADS MEF2-like
*BoSOC1.1.C4*	Bo4g024850	213	C4: 4425685–4,428,128 (−)	C4: 4021498–4023,941(−)	MADS MEF2-like
*BoSOC1.2.C4*	Bo4g195720	213	C4: 56667209–56,669,626 (−)	C4: 53015176–53,017,593(−)	MADS MEF2-like
*BoLFY.C2*	Bo2g161690	419	C2: 54420229–54,422,778 (+)	C2: 50956976–50,959,525(+)	Floricaula/Leafy protein, MADS-box, SAM domain, DNA-binding C-terminal domain
*BoCO.C1*	Bo1g105550	386	C1:34487516-34,488,935 (−)	C1: 32389932–32,391,351 (−)	B-box type zinc finger protein with CCT domain
*BoCO.C3*	Bo3g143010	416	C3:55639220-55,641,018 (−)	C3: 51379117–51,380,915 (−)	Zinc finger protein CONSTANS-LIKE 14
*BoCO.1.C4.*	Bo4g002240	373	C4:157832–159,255 (−)	C4: 153423–154,846 (−)	B-box type zinc finger protein with CCT domain
*BoCO.2.C4*	Bo4g156090	313	C4:45306026–45,307,048 (+)	C4: 42763759–42,764,781 (+)	zinc finger protein CONSTANS-LIKE 3
*BoCO.C5*	Bo5g073010	314	C5:26092051-26,093,117 (−)	C5: 24568350–24,569,416 (−)	B-box type zinc finger protein with CCT domain
*BoCO.C9*	Bo9g163730	366	C9:51326692-51,327,979 (+)	C9: 48282585–48,283,872 (+)	B-box type zinc finger protein with CCT domain
*BoVRN1.C1*	Bo1g115980	343	C1:37054708–37,057,372 (+)	C1: 34825349–34,828,013 (+)	B3 domain-containing transcription factor VRN1
*BoVRN2.C8*	Bo8g045980	437	C8:16005186–16,007,664 (−)	C8: 15423858–15,426,336 (−)	VEFS-Box of polycomb protein
*BoVIN3.C3*	Bo3g095330	428	C3:38317563-38,318,923 (+)	C3: 34746716–34,748,076 (+)	Oberon, PHD finger domain
*BoSVP.C4*	Bo4g149800	241	C4:43101011–43,103,883 (+)	C4: 40723422–40,726,294 (+)	MADS-box protein SVP-like
*BoSVP.C8*	Bo8g101000	241	C8:37659626–37,662,686 (−)	C8: 35302475–35,305,535 (−)	MADS-box protein SVP
*BoSPL.C2*	Bo2g062840	333	C2:20199281–20,200,431 (+)	C2: 18471026–18,472,176 (+)	Squamosa promoter-binding-like protein 6 isoform X1
*BoSPL.C4*	Bo4g042390	157	C4:10309157–10,309,713 (−)	C4: 9471445–9,472,001 (−)	Squamosa promoter-binding-like protein
*BoSPL.1.C6*	Bo6g029290	183	C6:7117888–7,118,508 (+)	C6: 6726669–6,727,289 (+)	Squamosa promoter-binding-like protein
*BoSPL.2.C6*	Bo6g031220	179	C6:7920394–7,921,332 (+)	C6: 7482570–7,483,508 (+)	Squamosa promoter-binding-like protein
*BoSPL.C8*	Bo8g118210	335	C8:44900056–44,901,763 (+)	C8: 41670596–41,672,303 (+)	Squamosa promoter binding protein-like protein 8

### Analysis of sequences and similarity of *BoFLC*, and integrator genes

The structures of the selected twenty five genes were accomplished to know their protein length, locations on chromosomes, and distribution of domains (Table 1). Our analysis showed that the genes contained; MADS-box, K-box, MADS MEF2-like, MADS AFFECTING FLOWERING 5-like isoform X1, Phosphatidylethanolamine-binding protein, Floricaula/Leafy protein, SAM, DNA-binding C-terminal, B-box type zinc finger protein with CCT, CONSTANS-LIKE 14, CONSTANS-LIKE 3, B3 domain-containing transcription factor VRN1, VEFS-Box of polycomb protein, and Squamosa promoter-binding-like protein domains. We also searched Ensembl Plants (https://plants.ensembl.org/index.html) to identify the ortholog sequences of these genes, and calculated their percent identity, query coverage, and gene order conservation (GOC) with *A. thaliana* and *B. napus* (Additional file 1: Table S1).

### Detection of DNA polymorphism in the selected gene

**Fig. 1:**
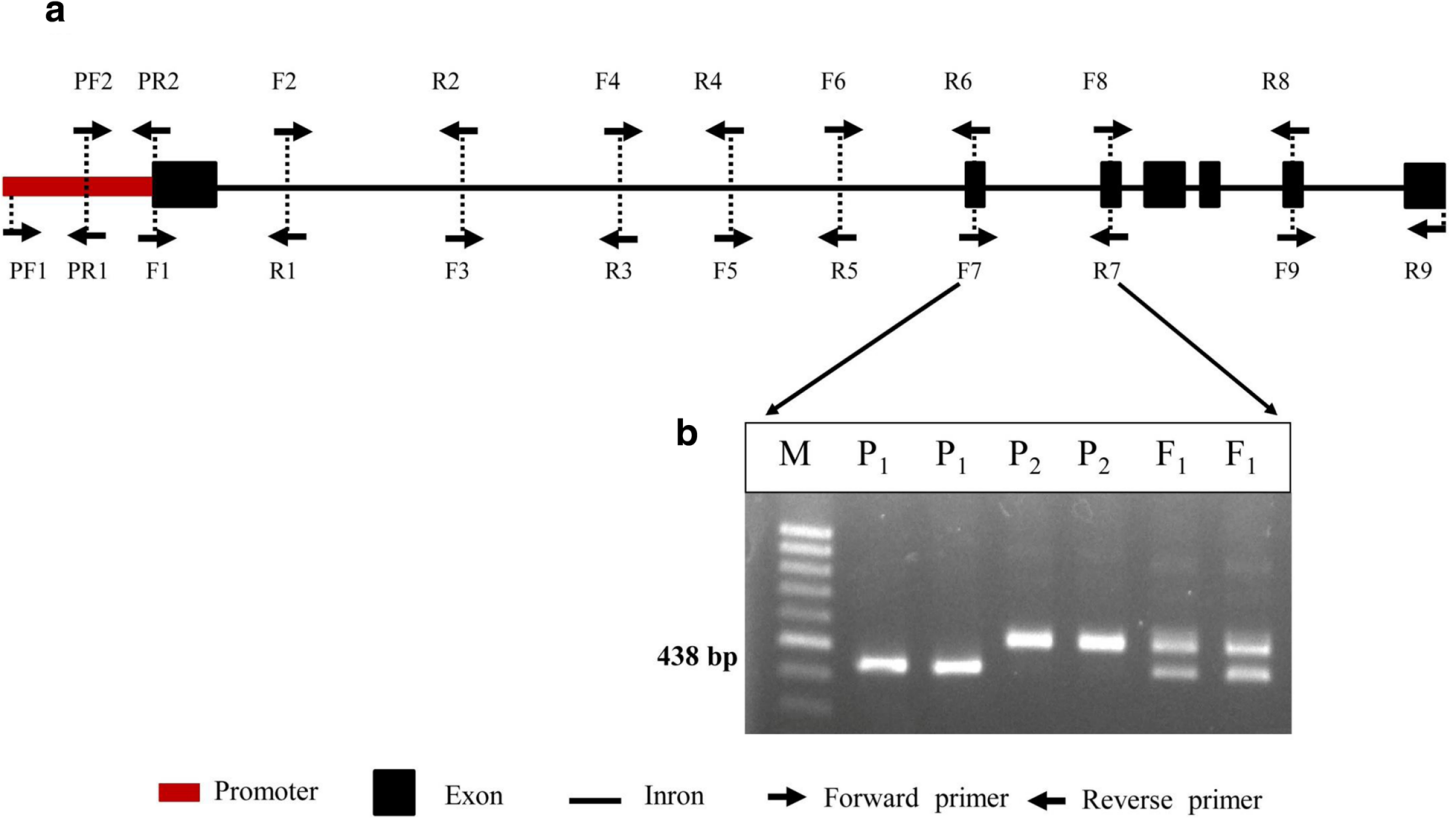
Gene structure of *BoFLC1.C9*, in which different primers are designed to detect polymorphism. **a** Schematic representation of the positions of primers used to detect polymorphism covering the promoter to stop codon region. Red box, promoter; black box, exon; black line, intron; forward arrow, forward primer; reverse arrow, reverse primer. **b** Polymorphic PCR amplicons with the F7R7 marker for the late-flowering line (P_1_) = BN3848 and the early-flowering line (P_2_) = BN623, as well as their F_1_ hybrid. M: 100 bp DNA marker (bioD, Gwangmyaong, Korea)

PCR (polymerase chain reaction) was used to amplify gene-specific forward and reverse primer pairs covering the promoter (1000 bp at the 3′ UTR; untranslated region) to the stop codon of each of the genes (*BoFLC*, *BoFT*, *BoSOC1*, *BoLFY*, *BoCO*, *BoVRN*, *BoSVP*, and *BoSPL*). Different combinations of the designed primer pairs were used to detect any size polymorphism in PCR amplicons between early- and late-flowering cabbage lines. However, no size polymorphism was detected for most of the genes (Additional file 2: Figure S1 and Additional file 3; for *BoFLC* genes as representation), except in *BoFLC1.C9*. Further, we analyzed details of the *BoFLC1.C9* gene to confer the nature of polymorphism. By using primer sets F7R7 with expected product sizes of 438 bp, revealed distinct size polymorphisms with insertion/deletion (Indel) mutations in the second intron (Fig. 1, Table 2).

**Table 2:** List of primers used to follow the mutation of *BoFLC1.C9* gene in parental lines, F_1_ and segregating F_2_ by polymerase chain reaction (PCR); and relative expression analysis of the target and *Actin* genes in quantitative PCR (qPCR)

Gene name	Accession number	Primer code	Sequences (5′→3′)	Product size (bp)	Marker type	References
*BoFLC1.C9*	Bo9g173400	F7: R7:	GGAAAGCAACATGGTGATGA CATGGTGTGAACCAGAGTCC	438	*Indel*	Present study
		F: R:	CTCTACAGCTTCTCCTCCGG TGTGAACCAGAGTCCAAAGC	119	qPCR	Present study
*Actin*	AF044573	1F: 1R:	TTCTCTCTTCCACACGCCAT CTTGTCCTGCGGGTAATTCG	235		[62]
	JQ435879	2F: 2R:	GTCGCTATTCAAGCTGTTCTCT GAGAGCTTCTCCTTGATGTCTC	251		[63]
	XM_013753106	3F: 3R:	ATCACACTTTCTACAATGAGC TCGTAGATTGGCACAGTGTGAG	241		[64]

### Cloning, sequencing, and sequence alignment

PCR amplicons from early- and late-flowering lines were cloned, sequenced and aligned to detect the number and position of the sequence polymorphism. Alignment of cloned sequences against the reference genes confirmed the presence of a 67-bp insertion in the second intron of early-flowering BN623 line at 2711–2712 bp position only for *BoFLC1.C9* (Additional file 2: Figure S2).

**Fig. 2:**
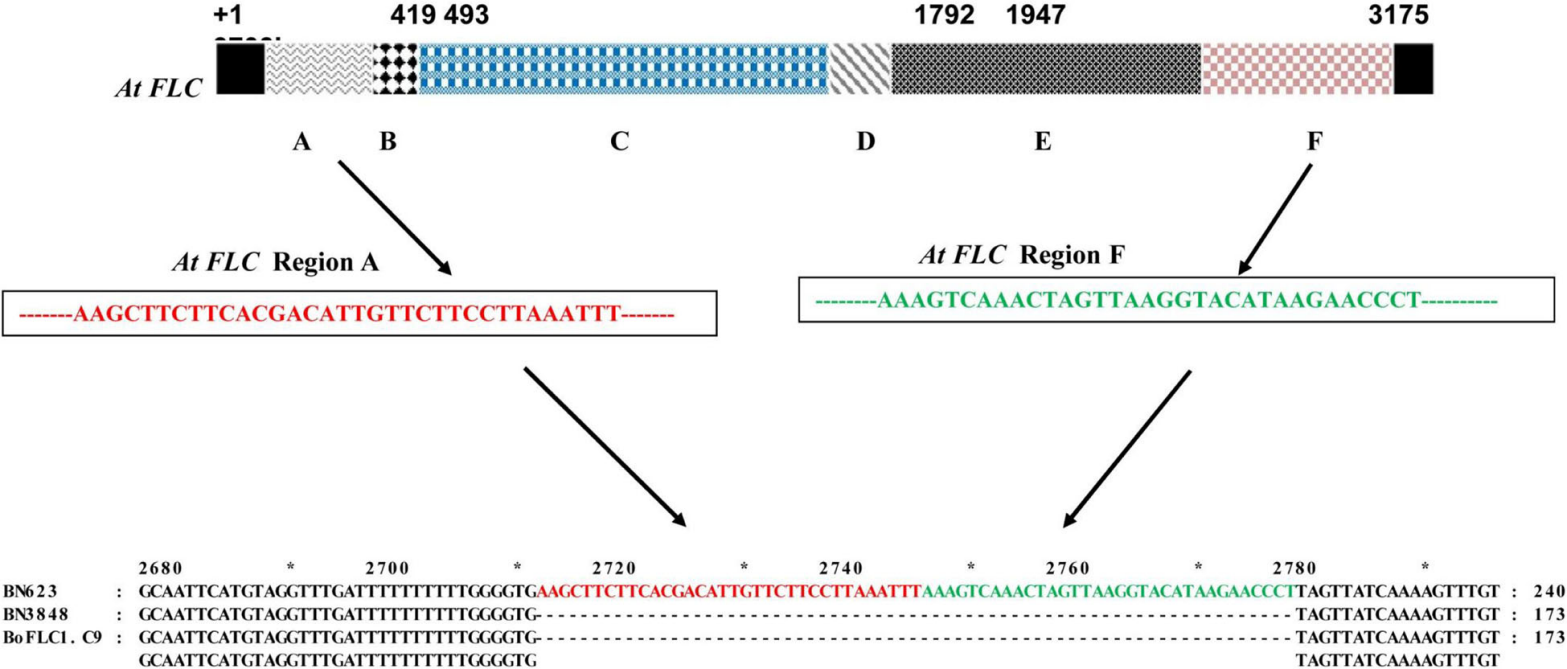
Pictogram of intron 1 from *Arabidopsis thaliana FLC* (*AtFLC*) and sequence alignment of *BoFLC1.C9* in the early-flowering line BN623. Red-colored (*AtFLC* region A) and green-colored (*AtFLC* region F) sequences are conserved in the inserted portion. Exons are shown in black. Intron 1 from *AtFLC* is partitioned to show the regulatory segments involved in various *FLC* activities [30]. Segments A and F are essential for down-regulation of the gene and other segments are maintained the regulatory system. Sixty-seven base pair-long segments inserted into intron 2 of *BoFLC1.C9* are shown in red and green; + 1 indicates the start codon (ATG)

This mutation of the target gene may have altered gene expression and flowering time. We analyzed the second intron of *BoFLC1.C9* (Bo9gl73400) because of the presence of a 67 bp insertion in the second intron in the early-flowering line BN623. We found inserted conserved segments of ‘A’ and ‘F’ of intron 1 of *AtFLC* sequence, of which contains the six segments A – F, in the second intron of *BoFLC1.C9* (Fig. 2), which reported as the suppressor of *FLC* gene [30] and produce early-flowering.

### Intron sequence variation interferes with gene expression

**Fig. 3:**
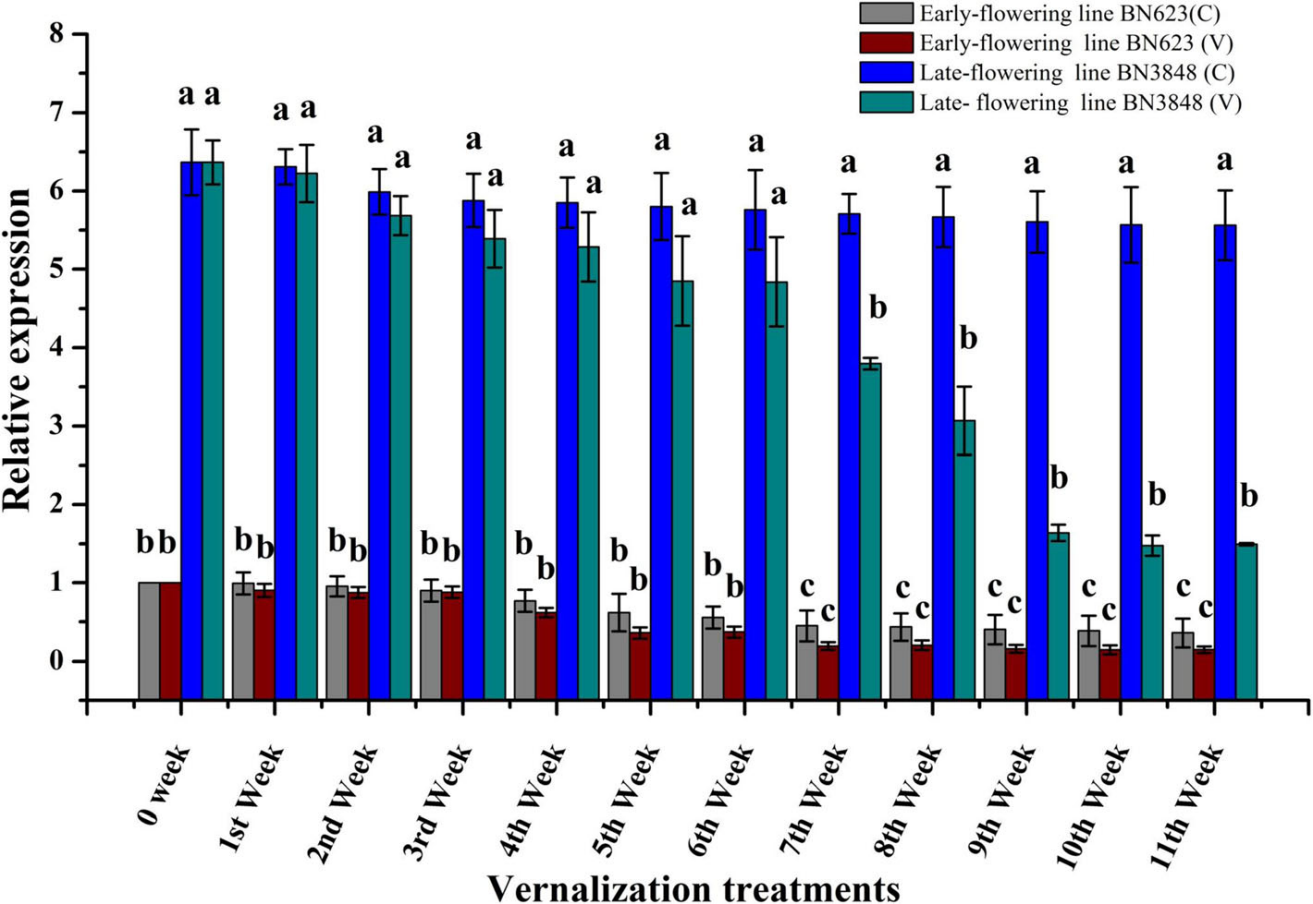
Relative expression of *BoFLC1.C9* using cDNA from vernalized (V) and non-vernalized (C: control) early-flowering line BN623 and late-flowering line BN3848. Bars indicate the mean values of five replications ± SD. Different lower case letters indicate statistically significant differences at *p* < 0.05 according to Tukey’s method

To understand the effect of vernalization on early- and late-flowering lines, the relative gene expression of *BoFLC1.C9* (Bo9gl73400) was estimated by qPCR using cDNA synthesized from leaf samples of pre-vernalized (0 week), weekly vernalized plants (1–11 week) and the samples of control plants (without vernalization). In both early- and late-flowering lines, expression of *BoFLC1.C9* (Bo9gl73400) decreased with increasing duration of vernalization. However, the expression of the gene in the early-flowering line was significantly lower (at least 5 times) at any given time point compared to the late-flowering line. In fact, even after 11 weeks of vernalization, the expression of this gene in the late-flowering line was never as low as in the pre-vernalized early-flowering line. In the early- and late-flowering lines, expression started to decline significantly after 3 and 6 weeks of vernalization, respectively (Fig. 3). In case of control (without vernalization), gene expression was non-significantly declined with the advance of time points (0–11 weeks) for both of the early- and late-flowering lines. In addition, unresponsiveness to the vernalization of the early-flowering line confirmed by the non-significant difference in gene expression between vernalized and non-vernalized plants at each time point. We concluded that a 67 bp insertion in the second intron may cause a loss of normal function and lower expression of *BoFLC1.C9*.

### Characterization of flowering time with proposed markers in the F_2_ population

**Fig. 4:**
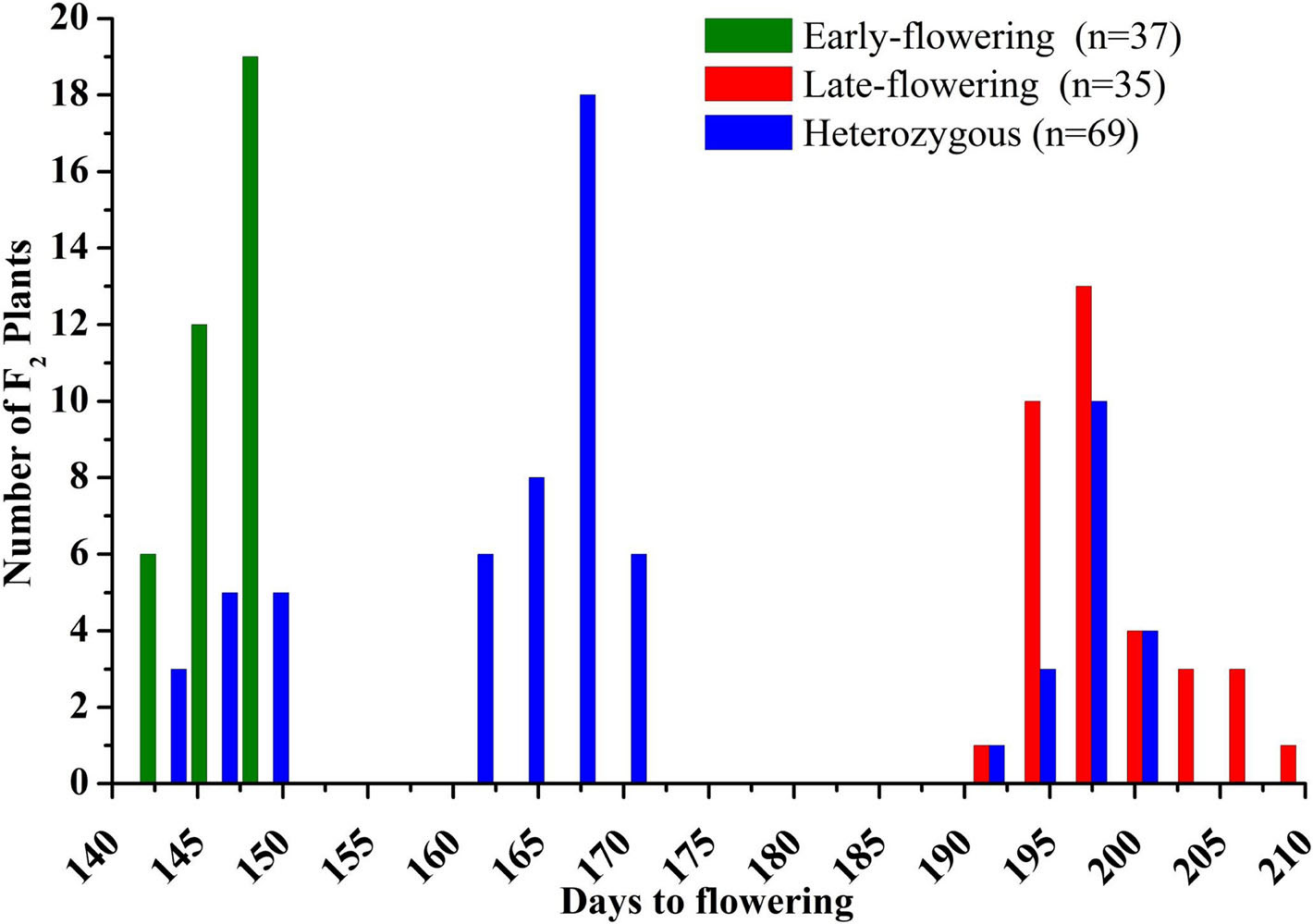
Frequency distribution of phenotypes and genotype of ‘F7R7’ marker linked to flowering time variations in F_2_ individuals

**Fig. 5:**
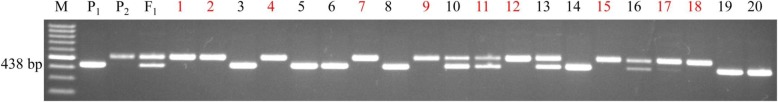
Markers are validated with 20 commercial early- and late-flowering cabbage lines, with ‘F7R7’ marker; of *BoFLC1.C9* gene matched 80% with the lines, the heterozygous lines (lanes: 10, 11, 13, and 16) are not matched with the phenotypes exactly. Here, P_1_ = late-flowering parent (BN3848), P_2_ = early-flowering parent (BN623), F_1_ = (BN3848 × BN623), M = 100 bp DNA marker, Red color numbered (10 early-flowering lines): lane 1, 2, 4, 7, 9, 11, 12, 15,17 are 17 FLE1, 17 FLE2, 17 FLE3, 17 FLE4, 17 FLE5, 17 FLE6, 17 FLE7, 17 FLE8, 17 FLE9, and 17 FLE10, respectively and black color numbered (10 late-flowering lines): lanes 3, 5, 6, 8, 10, 13, 14, 16,19 and 20 are 17 FLL1, 17 FLL2, 17 FLL3, 17 FLL4, 17 FLL5, 17 FLL6, 17 FLL7, 17 FLL8, 17 FLL9, and 17 FLL10, respectively

We recorded the flowering times, which were 140 to 150 days after sowing (DAS) for the early-flowering line BN623 and ≥ 190 DAS for the late-flowering line BN3848, while flowering times were varied from 142 to 210 DAS in the F_2_ generation (Fig. 4). Among the F_2_ individuals, fraction of heterozygous plants (presence of both parental bands) showed flowering time variations between 143 to 200 DAS. Our proposed marker ‘F7R7’ can explained 83% of the phenotypic variations of 141 F_2_ individuals regarading DAS (Additional file 2: Figure S3 and S4). While, 20 commercial lines showed 80% match with this marker considering the PCR amplification of early- and late-flowering lines (Fig. 5). The marker ‘F7R7’ (Table 2) was designed on the *BoFLC1.C9* gene, which was able to explain 83% phenotypic variations of the early- and late-flowering corresponded to PCR amplifications in the F_2_ generation (Additional file 2: Figure S3 and S4).

## Discussion

Flowering is a multipart physiological characteristic controlled by a series of integrator genes, vernalization genes, and by the plant spending it’s vegetative phase exposed to cold temperatures for a certain period. It is necessary to develop molecular markers to select early-and late-flowering cabbage lines before sowing from a breeding population. Overwinter types must be exposed to cold (vernalization) to transition from vegetative growth to flowering, but this is not obligatory for spring varieties, which are generally grown in areas with shorter seasons. Many genetic and environmental factors are involved in this transition, and signals of these factors are integrated into the causal effect of flowering [31]. Transition of flowering from vegetative phase is the resultant of the interactions between transducer proteins and integrator signals, which either promote or inhibit the transition process [10, 32]. In *Arabidopsis*, the key flowering genes have been identified and characterized [6, 33]. Flowering genes are involved in a ‘floral integrator’ network, comprised with six regulatory gene pathways [31]. *FLC* is a key member of the floral integrator network, and its expression is influenced by low temperature.

There are multiple *FLC* and ‘floral integrator’ homolog genes in cabbage (*B. oleracea* var. *capitata*) were duplicated before divergence of *Brassica* from their ancestor [21]. The group of genes with MADS-box domains consists of five MADS AFFECTING FLOWERING (MAF) proteins [34]. *FLOWERING LOCUS M* (*FLM*, known as *MAF1*) represses flowering [35, 36], through vernalization dependent repression of *FLM* and *MAF1* genes and accelerates flowering [37–39].

Among the selected 25 genes (3 *BoFLC*, 2 *BoFT*, 3 *BoSOC1*, 1 *BoLFY*, 6 *BoCO*, 3 *BoVRN*, 2 *BoSVP*, and 5 *BoSPL*), we found DNA size polymorphisms only in *BoFLC1.C9* (Bo9gl73400) gene between early- and late-flowering lines by using several primers at different positions. The polymorphic *BoFLC1.C9* (Bo9gl73400) gene contains MADS-box and K-box domain proteins, thus it might be involved in flowering time variation in cabbage. By contrast, in the *Brassicaceae*, suppression of flowering is mediated by vernalization, which mainly involves major-effect changes at a few loci [40–43]. Of these, *FLC* appears to be the predominant source of variation in the vernalization response [44, 45], and ultimately causes the variation in flowering time. Association and/or quantitative trait locus (QTL) mapping corroborated that variation in flowering time of *B. oleracea*, *B. rapa*, and *B.napus* is linked to polymorphism in *FLC* homologs and responded upon vernalization [46–49].

In *BoFLC1.C9* (Bo9gl73400), the ‘F7R7’ marker (Table 2) showed polymorphism across intron 2. We linked this mutation with flowering time variation in the F_2_ population of the cross (BN3848 (late-flowering) × BN623 (early-flowering)) and twenty commercial cultivars. *FRI* gene was identified as a major determinant of flowering time variation in *A. thaliana* population through it’s effect on *FLC* [50, 51]. In addition, *indel* polymorphism of *CONSTANS LIKE 1* (*COL1*) has also been associated with flowering time variation in *B. nigra* [52].

In this study, lower relative expression of the *BoFLC1.C9* gene was found in the early-flowering line, where presence the *Indel* variation, compared to the late-flowering line (Fig. 3). The presence of spliced long noncoding RNA in *COOLAIR* locus of late-flowering line leading to higher *FLC* expression and delay flowering. This is supported by the identification of a single nucleotide polymorphism (SNP) in *A. thaliana* haplotype which caused splicing of long noncoding RNA (lnRNA) at *COOLAIR* locus leading to higher *FLC* expression and increased requirement for vernalization or delayed flowering [38]. *COOLAIR* splicing disrupted COOLAIR production due to splice at the *COOLAIR* proximal acceptor site [53] and suggested that splicing of lnRNA could be modulated *FLC* expression quantitatively through co-transcriptional coupling mechanisms [53]. Conservation of COOLAIR [54] and presence of the *AtFLC* antisense RNA in *Arabis alpine* [55] and *B. rapa* [56] have been pointed as strong commonality in the regulation of *FLC* across the *Brassicaceae*.

Noncoding sequence alteration has recently been identified in *AtFLC* haplotype groups with different degree of *AtFLC* expression and epigenetic silencing [57]. In *B. oleracea*, one of two major *FLC* haplotypes is transcriptionally repressed by exposure to cold more slowly than others [46]. Intron 2 of the *BoFLC1.C9* gene might be responsible for *FLC* repression activities by accommodating segments of intron 1 of *Arabidopsis FLC* (Fig. 2). The intron 1 of *Arabidopsis FLC* segments are required to maintain *FLC* repression [30]. The segregating F_2_ population showed wide range of variation in flowering time followed neither in continuous variation nor in Mendelian inheritance (Fig. 4).

Results obtained from DNA size polymorphism analysis of *BoFLC1.C9* and the F_2_ phenotypes suggest that *BoFLC1.C9* is a likely candidate for causing variation in flowering time in this population. It has previously been reported that regulation of *FLC* is controlled by the regions in its intron [30, 58], with polymorphisms leading to differential expression and splicing patterns. The proposed marker ‘F7R7’ based on 67 bp insertion in intron 2 accounted for 83% of flowering time variation, among the F_2_ population. While, in commercial lines ‘F7R7’ makers matched up to 80%. Ridge et al. [15] found 65% flowering time variation in the F_2_ population of a cross of late- and early-flowering cauliflower lines. A 67 bp insertion in the second intron of *BoFLC1.C9* gene in the early bolting line made a distinct mutation and disrupted the function of the gene and showed lower expression caused early-flowering.

## Conclusions

Using molecular markers and relative expression-based approaches, we reported the sequence variations in *BoFLC1.C9* gene for characterizing early- and late-flowering cabbage lines. Our result suggests that naturally occurring ‘*Indel*’ confirmed by ‘F7R7’ marker in intron 2 in the *BoFLC1.C9* gene is able to characterize early- and late-flowering cabbage lines up to 80% variation. This marker might be useful for selecting desired early- and/or late-flowering cabbage cultivars before cultivation. Further experiments on presence of copy number of *BoFLC1.C9* gene could elucidate the mismatched fractions of the ‘F7R7’ marker more clearly.

## Methods

### Plant materials and evaluation of flowering time

Two inbred lines (late-flowering BN3848 and early-flowering BN623) with distinct flowering times varying by 40–45 days, were used to develop F_1_ and F_2_ generation plants by crossing and selfing, respectively. The average flowering time was 140 to 150 DAS for the early-flowering line BN623 and ≥ 190 DAS for the late-flowering line BN43848. The F_1_ generation was designated as intermediate flowering with 160–175 DAS. Whereas, twenty commercial cabbage cultivars collected from Sunchon National University Cabbage breeding germplasm, which were characterized as early-flowering (140–150 DAS) and late-flowering (≥195 DAS). A pot-based glasshouse trial was conducted at Sunchon National University, South Korea using five plants of parental lines, five from the F_1_ generation, and 141 from the F_2_ generation to record variations in flowering time. Seeds of the plant materials were germinated in a multipot tray using cocopit soil, and were allowed to grow in a growth chamber at 24 °C, 60% relative humidity, and under 16 h/8 h (light/dark) conditions for up to 60 days. Then, in September 2015, eight weeks old plants at 8 leaf stage were transferred to larger pots (30 × 25 cm) filled with a mixture of 50% cocopit and 50% soil. Plants were allowed to overwinter inside the glasshouse, where temperature and day length were recorded in the range of − 5 °C to 8 °C with 10 h/14 h (light/dark) during the winter and 12–17 °C with 12 h/12 h (light/dark) during the spring. The number of days to flowering was recorded as the day on which the first flower of individual plants was observed to open after seedlings were transplanted to larger pots in the glasshouse. Plants that did not flower within 190 days were considered to be late flowering.

### Gene selection and sequence analyses

The flowering pathway gene sequences of *A. thaliana* were collected from TAIR (https://www.arabidopsis.org/) and the syntenic genes of *B. oleracea* were collected from the BRAD database (http://brassicadb.org/brad/) using syntenic gene search and cross-checked against the Bolbase (http://www.ocri-genomics.org/bolbase/genes.htm) as well as EnsemblPlants (http://plants.ensembl.org/) databases. A complementary method, Hidden Markov Models (HMM) profiling was performed using the Bolpangenome (http://www.brassicagenome.net/) database to increase the accuracy of the identified genes. The National Center for Biotechnology Information (NCBI) (https://www.ncbi.nlm.nih.gov/Structure/cdd/wrpsb.cgi) web tool was used for searching the domains.

### Isolation of DNA and detection of DNA polymorphism

The protocol described by Ishizawa et al. [59] with slight modifications was followed for extracting DNA from four weeks leaf samples of parental lines, five F_1_ plants, 141 F_2_ plants, and 20 commercial cultivars. Primer3Plus online tool was used for designing locus-specific primers. The newly designed and previously reported primers [15, 22, 46, 60] (listed in Table 2, and Additional file 1: Table S2A,B) were used to identify DNA polymorphisms between the contrasting lines. A total volume of 20 μl was used in PCR, which contained 1 μl DNA (80 ng), 1 μl (10 pmol) of forward and 1 μl (10 pmol) of reverse primers, 8 μl Prime Taq-premix (2×) (GENETBIO Inc., Gwangmyaong, Korea), and 9 μl ultra-pure H_2_O. PCR was carried out in a thermo-cycler set as 5-min initial denaturation at 95 °C, followed by 30 cycles of denaturation at 95 °C for 1 min, annealing at 58 °C for 1 min, elongation at 72 °C for 1 min, final elongation at 72 °C for 10 min, and cool down at 4 °C. PCR products were separated in 2% agarose gel stained with HiQ blue mango (20,000×) (bioD, Gwangmyaong, Korea) and visualized with ultraviolet light.

### Cloning and sequencing of the polymorphic gene

Promega Purification Kit (Promega, Madison, WI, USA) was used for purification of the amplified DNA fragments following manufacturer’s instructions. TOPO TA Cloning Kit (Invitrogen, Carlsbad, CA, USA) was used for cloning. Three independent clones were separated from the PCR amplicon of the polymorphic gene, amplified in both early- and late-flowering lines. The universal primers M13F and M13RpUC were used for sequencing the cloned DNA by using ABI3730XL sequencer (Macrogen Co., Seoul, South Korea). Cloned sequences were aligned with a reference sequence using ClustalOmega [61] to identify the types and positions of sequence variations.

### Isolation of RNA, synthesis of cDNA and expression profiling of the polymorphic gene

The RNeasy Mini Kit (Qiagen, Hilden, Germany) was used for isolating total RNA from leaf samples collected from the different levels of vernalized plants, and total RNA was purified with a Qiagen RNase-free DNase1 Kit (Qiagen, Hilden, Germany). For the vernalization treatment, a set of plants at 8th leaf stage was transferred to incubators (TOGA clean system; model: TOGA UGSR01, Daejong, Korea) maintained at 4 °C with 14 h/10 h (light/dark) until 11 weeks of vernalization. Leaf samples were excised from five replicated plants of prevernalized (0 week), 1-, 2-, 3-, 4-, 5-, 6-, 7-, 8-, 9-, 10-, and 11- week of vernalization and control (no vernalization) as checked. The leaf samples were immersed quickly in liquid nitrogen and stored at − 80 °C to avoid degradation of RNA. The RNA was quantified by using NanoDrop® 1000 Spectrophotometer (Wilmington, DE, USA) and 6 ng of RNA per sample was used for synthesizing first-strand cDNA by using the Superscript®III First-Strand cDNA Synthesis Kit (Invitrogen, Carlsbad, CA, USA) with oligo-dT primer. Gene-specific primers of the candidate *FLC* (*BoFLC1.C9*) and *Actin* genes were used for qPCR (Table 2). A total volume 10 μl PCR mastermix for each sample contained 1 μl of cDNA (70 ng), 1 μl (10 pmol) of each forward and reverse primer, 2 μl double distilled water, and 5 μl Taq™ from the SYBR®Green PCR Kit (ThermoFisher, California, USA)of was used for conducting qPCR. A Lightcycler®96SW 1.1 (Roche, Dusseldorf, Germany) programmed as pre-denaturation at 95 °C for 5 min, followed by 40 cycles of denaturation at 94 °C for 10 s, annealing at 58 °C for 10 s, and extension at 72 °C for 15 s was used for carrying out the qPCR. Gene expression levels for each sample were normalized by using the average ‘Ct’ value of the 3 *Actin* genes as a reference. The 2^−∆∆Ct^ method was used to calculate relative expression [34]. One-way analysis of variance (ANOVA) and mean separations of the relative expression of genes were calculated using with MINITAB version 18 statistical software (Minitab Inc., State College, PA, USA).

## Additional files

Additional file 1: **Table S1.** Ortholog species, ortholog ID, percent identity, percent query coverage and GOC of 25 genes of *BoFLC*, *BoFT*, *BoSOC1*, *BoLFY*, *BoCO*, *BoVRN*, *BoVIN*, *BoSVP* and *BoSPL* of *B. oleracea*. **Table S2** A. List of newly designed primers on the identified 25 genes used for searching polymorphism by PCR. **Table S2** B. List of previously published primers on the reported *FLC*s in *B. oleracea* used in this study. (DOC 272 kb)

Additional file 2: **Figure S1.** PCR amplicons of *BoFLC1.C9*, *BoFLC3.C3* and *BoFLC4.C3* in late-flowering line BN3848 (P_1_) and early-flowering line BN623 (P_2_). PCR products with respective primers from start to stop codons of the genes (Additional file 1: Table S1A) were run on a 1.5% agarose gel and their corresponding amplicon sizes are mentioned. M is a 100-bp size marker. **Figure S2.** Sequence alignments of the *BoFLC1.C9* gene cloned from early- and late-flowering lines. Variation of a 67-bp insertion in the early-flowering line BN623 is highlighted in red color. **Figure S3.** PCR-amplicons of 141 F_2_ segregating population with F7R7 primers of Indel marker of *BoFLC1.C9* gene. P_1_ = Late-flowering parent (BN3848), P_2_ = Early-flowering parent (BN623), F_1_ = (BN3848 × BN623); black and red colored numbers of the F_2_ individual are matched and mismatched lines, respectively as early- and late-flowering lines. M = 100 bp DNA marker. **Figure S4.** Regression and correlation coefficient between marker dosage and phenotypes as days to flowering after sowing (DAS) explained by the F7R7 marker in 141 F_2_ individual. ** indicates *p < 0.01*. (PPT 2295 kb)

Additional file 3: Sequence alignment of the total sequences of the gene *BoFLC3.C3* and *BoFLC4.C3* cloned from early-flowering line (BN623) and late-flowering line (BN3848) aligned with reference sequence of *BoFLC3.C3* and *BoFLC4.C3* genes, respectively. Red highlighted letter indicate SNPs variations in the early-flowering line. Forward and reverse arrows indicate forward and reverse primer sets used for cloning and sequencing. (DOC 747 kb)

## Abbreviations

*COL1*: *CONSTANS LIKE 1*
DAS: Days after sowing
*FLC*: *FLOWERING LOCUS C*
*FLM*: *FLOWERING LOCUS M*
*FT*: *FLOWERING LOCUS T*
GOC: Gene order conservation
*Indel*: Insertion/deletion
*LFY*: *LEAFY*
*MAF*: *MADS AFFECTING FLOWERING*
PAVs: Presence/absence variants
PCR: Polymerase Chain Reaction
SD: Standard deviation
*SOC1*: *SUPPRESSOR OF OVEREXPRESSION OF CONSTANS1*
*SPL*: *SQUAMOSA PROMOTER BINDING LIKE*
*SVP*: *SHORT VEGETATIVE PHASE*
UTR: Untranslated region
*VRN*: *VERNALIZATION*
