# Supplementary material for: Molecular markers based on sequence variation in BoFLC1.C9 for characterizing early- and late-flowering cabbage genotypes
Source: BMC Genet. 2019 Apr 27;20:42. doi: 10.1186/s12863-019-0740-1 (PMC6487051; doi:10.1186/s12863-019-0740-1)
Supplement: Supplementary file 1 — Table S1. Ortholog species, ortholog ID, percent identity, percent query coverage and GOC of 25 genes of BoFLC, BoFT, BoSOC1, BoLFY, BoCO, BoVRN, BoVIN, BoSVP and BoSPL of B. oleracea. Table S2 A. List of newly designed primers on the identified 25 genes used for searching polymorphism by PCR. Table S2 B. List of previously published primers on the reported FLCs in B. oleracea used in this study. (DOC 272 kb) [file 12863_2019_740_MOESM1_ESM.doc]

**Table S1.** Ortholog species, ortholog ID, percent identity, percent query coverage and GOC of 25 genes of *BoFLC*, *BoFT*, *BoSOC1*, *BoLFY*, *BoCO*, *BoVRN*, *BoVIN*, *BoSVP* and *BoSPL* of *B. oleracea*.

| Gene ID | *Ortholog species* | Ortholog ID | Identity (%) | Query coverage (%) | GOC score |
| --- | --- | --- | --- | --- | --- |
| *BoFLC1.C9* | *A. thaliana* | FLC  (AT5G10140) | 85.71 | 85.28 | 50 |
|  | *B. napus* | BnaC09g46540D | 85.59 | 99.49 | 75 |
| *BoFLC3.C3* | *A. thaliana* | FLC  (AT5G10140) | 83.67 | 83.25 | 25 |
|  | *B. napus* | BnaC03g04170D | 98.98 | 98.98 | 100 |
| *BoFLC4.C3* | *A. thaliana* | AT5G65070 | 59.05 | 68.50 | 25 |
|  | *B. napus* | BnaA06g24000D | 93.53 | 94.00 | 75 |
| *BoFT.C5* | *A. thaliana* | MFT  (AT1G18100) | 94.22 | 93.68 | 50 |
|  | *B. napus* | BnaC05g50120D | 99.43 | 99.43 | 50 |
| *BoFT.C8* | *A. thaliana* | MFT  (AT1G18100) | 95.38 | 95.38 | 50 |
|  | *B. napus* | BnaC08g37400D | 100.00 | 100.00 | 100 |
| *BoSOC1.C3* | *A. thaliana* | SOC1  (AT2G45660) | 92.99 | 93.43 | 75 |
|  | *B. rapa* | Bra000393 | 96.24 | 96.24 | 75 |
| *BoSOC1.1.C4* | *A. thaliana* | SOC1  (AT2G45660) | 94.86 | 95.31 | 50 |
|  | *B. rapa* | Bra004928 | 98.12 | 98.12 | 100 |
| *BoSOC1.2.C4* | *B. napus* | BnaC04g50370D | 98.12 | 98.12 | 75 |
|  | *B. rapa* | Bra039324 | 98.59 | 98.59 | 100 |
| *BoLFY.C2* | *B. rapa* | Bra029305 | 98.80 | 97.85 | 100 |
|  | *A. thaliana* | LFY  (AT5G61850) | 86.08 | 87.11 | 0 |
| *BoCO.C1* | *Brassica rapa* | [Bra023858](http://plants.ensembl.org/Brassica_rapa/Gene/Summary?g=Bra023858) | 97.41 | 97.41 | 100 |
|  | *Brassica napus* | BnaCnng43560D | 99.46 | 95.60 | 75 |
| *BoCO.C3* | *Brassica napus* | BnaC03g58230D | 100.00 | 94.47 | 100 |
|  | *Arabidopsis thaliana* | COL15  [(AT1G28050)](http://plants.ensembl.org/Arabidopsis_thaliana/Gene/Summary?g=AT1G28050) | 74.36 | 77.40 | 25 |
| *BoCO.1.C4.* | *Brassica rapa* | [Bra004423](http://plants.ensembl.org/Brassica_rapa/Gene/Summary?g=Bra004423) | 95.51 | 85.52 | 100 |
|  | *Brassica napus* | BnaC04g00040D | 100.00 | 89.54 | 75 |
| *BoCO.2.C4* | *Brassica rapa* | [Bra032061](http://plants.ensembl.org/Brassica_rapa/Gene/Summary?g=Bra032061) | 93.77 | 86.58 | 100 |
|  | *Brassica napus* | BnaC04g37230D | 98.97 | 91.69 | 75 |
| *BoCO.C5* | *Arabidopsis thaliana* | [AT1G49130](http://plants.ensembl.org/Arabidopsis_thaliana/Gene/Summary?g=AT1G49130) | 59.51 | 61.78 | 50 |
|  | *Brassica rapa* | [Bra032273](http://plants.ensembl.org/Brassica_rapa/Gene/Summary?g=Bra032273) | 63.06 | 63.06 | 0 |
| *BoCO.C9* | *Arabidopsis thaliana* | CO  [(AT5G15840)](http://plants.ensembl.org/Arabidopsis_thaliana/Gene/Summary?g=AT5G15840) | 70.51 | 71.86 | 100 |
|  | *Brassica napus* | BnaC09g41990D | 99.18 | 99.18 | 75 |
| *BoVRN1.C1* | *Brassica napus* | BnaC01g33680D | 99.42 | 99.42 | 100 |
|  | *Brassica rapa* | [Bra037544](http://plants.ensembl.org/Brassica_rapa/Gene/Summary?g=Bra037544) | 96.20 | 95.92 | 50 |
| *BoVRN2.C8* | *Arabidopsis thaliana* | VRN2  [(AT4G16845)](http://plants.ensembl.org/Arabidopsis_thaliana/Gene/Summary?g=AT4G16845) | 64.09 | 64.53 | 25 |
|  | *Brassica rapa* | [Bra021078](http://plants.ensembl.org/Brassica_rapa/Gene/Summary?g=Bra021078) | 93.65 | 91.08 | 0 |
| *BoVIN3.C3* | *Brassica napus* | BnaC03g74580D | 96.54 | 91.36 | 50 |
|  | *Brassica rapa* | [Bra024456](http://plants.ensembl.org/Brassica_rapa/Gene/Summary?g=Bra024456) | 83.99 | 84.58 | 100 |
| *BoSVP.C4* | *Brassica rapa* | [Bra030228](http://plants.ensembl.org/Brassica_rapa/Gene/Summary?g=Bra030228) | 98.31 | 96.27 | 100 |
|  | *Arabidopsis thaliana* | SVP  [(AT2G22540)](http://plants.ensembl.org/Arabidopsis_thaliana/Gene/Summary?g=AT2G22540) | 90.00 | 89.63 | 50 |
| *BoSVP.C8* | *Brassica napus* | BnaC08g34920D | 98.31 | 96.27 | 100 |
|  | *Arabidopsis thaliana* | SVP  [(AT2G22540)](http://plants.ensembl.org/Arabidopsis_thaliana/Gene/Summary?g=AT2G22540) | 92.08 | 91.70 | 75 |
| *BoSPL.C2* | *Brassica rapa* | [Bra038324](http://plants.ensembl.org/Brassica_rapa/Gene/Summary?g=Bra038324) | 79.14 | 77.48 | 50 |
|  | *Arabidopsis thaliana* | SPL6  [(AT1G69170)](http://plants.ensembl.org/Arabidopsis_thaliana/Gene/Summary?g=AT1G69170) | 38.77 | 47.15 | 25 |
| *BoSPL.C4* | *Brassica rapa* | [Bra005470](http://plants.ensembl.org/Brassica_rapa/Gene/Summary?g=Bra005470) | 93.66 | 84.71 | 100 |
|  | *Arabidopsis thaliana* | SPL3  [(AT2G33810)](http://plants.ensembl.org/Arabidopsis_thaliana/Gene/Summary?g=AT2G33810) | 80.15 | 66.88 | 75 |
| *BoSPL.1.C6* | *Arabidopsis thaliana* | SPL4  [(AT1G53160)](http://plants.ensembl.org/Arabidopsis_thaliana/Gene/Summary?g=AT1G53160) | 78.16 | 74.32 | 100 |
|  | *Brassica napus* | BnaC06g10070D | 98.09 | 84.15 | 50 |
| *BoSPL.2.C6* | *Arabidopsis thaliana* | SPL4  [(AT1G53160)](http://plants.ensembl.org/Arabidopsis_thaliana/Gene/Summary?g=AT1G53160) | 78.74 | 76.54 | 75 |
|  | *Brassica napus* | BnaC06g41420D | 98.88 | 98.88 | 50 |
| *BoSPL.C8* | *Arabidopsis thaliana* | SPL8  [(AT1G02065)](http://plants.ensembl.org/Arabidopsis_thaliana/Gene/Summary?g=AT1G02065) | 48.95 | 48.66 | 50 |
|  | *Brassica napus* | BnaCnng09040D | 99.70 | 99.70 | 100 |

**Table S2A.** List of newly designed primers on the identified 25 genes used for searching polymorphism by PCR.

| Gene Name | Forward (5'--------------3') | Reverse (5'---------------3') | Product Size (bp) | Primer Region |
| --- | --- | --- | --- | --- |
| *BoFLC1.C9* | PF1:GCCTAGAGTGCTAAGTGTCA | PR1:CATGTTTCTGTTGAGGTTGC | 448 | Promoter |
|  | PF2:GCAACCTCAACAGAAACATG | PR2:TCAAGTTTCTTTCTCCCCAT | 399 |  |
|  | F1:ATGGGGAGAAAGAAACTTGA | R1:CAAGTATGCATCACAGCGTG | 441 | 1st Exon -1st Intron |
|  | F2:CACGCTGTGATGCATACTTG | R2:CTTCGGATATCTTTTCGG | 533 | 1st Intron |
|  | F3:CCGAAAAGATATCCGAAG | R3:AGTGAGTCTGTGTTTTCCAG | 555 | 1st Intron |
|  | F4:CTGGAAAACACAGACTCACT | R4:CAACATGACCTGGGTCCAAC | 308 | 1st Intron |
|  | F5:GTTGGACCCAGGTCATGTTG | R5:AAACACAACGAGATGCAAGG | 432 | 1st Intron |
|  | F6:CCTTGCATCTCGTTGTGTTT | R6:TCATCACCATGTTGCTTTCC | 406 | 1st Intron- 2nd Exon |
|  | F8:GGACTCTGGTTCACACCATG | R8:TAGCCAAAACATGGTTCTCC | 610 | 3rd Exon-6th Exon |
|  | F9:GGAGAACCATGTTTTGGCTA | R9:CTAATTAAGCAGTGGGAGCG | 539 | 6th Exon-7th Exon |
| *BoFLC3.C3* | PF1: CCGGATCCGAAAAACCGAAC | PR1: AAATTTCGGTTCGGGTTCGG | 554 | Promoter |
|  | PF2: CCGAACCCGAACCGAAATTT | PR2: GAAGGTGACTTGTCGGCTAC | 447 |  |
|  | F1: GTAGCCGACAAGTCACCTTC | R1: GTGTTCTCTTTCGTTCCTCG | 949 | 1st Exon -1st Intron |
|  | F2: CGAGGAACGAAAGAGAACAC | R2: CCTATCCACAAGTTCAAGTA | 1136 | 1st Intron-3rd Exon |
|  | F3: TACTTGAACTTGTGGATAGG | R3: CTAATTAAGCAGTGGGAGAG | 1202 | 3rd Exon-7th Exon |
| *BoFLC4.C3* | PF1:CCTGTGACTATGGTAGGCTC | PR1: TAGGAATCTTTCAGTAGCAG | 511 | Promoter |
|  | PF2:CTGCTACTGAAAGATTCCTA | PR2: TCCACTTTTCTCCTTCCCAT | 529 |  |
|  | F1: ATGGGAAGGAGAAAAGTGGA | R1: TTCGTGTCTGTTAGCTTGCC | 954 | 1st Exon -1st Intron |
|  | F2: GGCAAGCTAACAGACACGAA | R2: AGCCTGCCTTACATTTTATC | 1180 | 1st Intron |
|  | F3: GATAAAATGTAAGGCAGGCT | R3: AGATATTAGAGACTGTACGG | 1054 | 1st Intron - 4th Exon |
|  | F4: CCGTACAGTCTCTAATATCT | R4: CTTGAGAAGCGGAAGAGTCT | 869 | 4th Exon-7th Exon |
| *BoFT.C5* | PF1:CCGGGTACGGATCGGTTCTT | PR1: GGATCAACCGAAGCTGCTGC | 859 | Promoter |
|  | F1: GCAGCAGCTTCGGTTGATCC | R1: CCTTTTGATGGATTGGTGCC | 713 | 1stExon–3rd Exon |
|  | F2: GGCACCAATCCATCAAAAGG | R2: CGTCTGCGTGAAGCTGGCTC | 905 | 3rd Intron - 4th Exon |
| *BoFT.C8* | PF1:GTGTGAAATGGCCTATGCAT | PR1: GGATCAACCGAAGCTGCCAT | 960 | Promoter |
|  | F1: ATGGCAGCTTCGGTTGATCC | R1: ACCAGTGGACCCATTCTCTC | 687 | 1st Exon- 2nd Exon |
|  | F2: GAGAGAATGGGTCCACTGGT | R2: GAGGGGTTGGTGCCTCCCGG | 637 | 2nd exon -3rd Exon |
|  | F3: CCGGGAGGCACCAACCCCTC | R3: CGTCTGCGTGAAGCTGGCTC | 897 | 3rd Intron - 4th Exon |
| *BoSOC1.C3* | PF1:CAGGAGACATAAACAAACCC | PR1: TGAGTTTTCCCCCTCACCAT | 920 | Promoter |
|  | F1: ATGGTGAGGGGGAAAACTCA | R1: AATATTAACCAGATCATTAC | 649 | 1st Exon -1st Intron |
|  | F2: GTAATGATCTGGTTAATATT | R2: CAAGATCACAATTAACCTGC | 721 | 1st Intron-2nd Intron |
|  | F3: GCAGGTTAATTGTGATCTTG | R3: CTTTCTTGCTCGAACACATT | 460 | 2nd Intron 4th Exon |
|  | F4: AATGTGTTCGAGCAAGAAAG | R4: AACAAGGTAACCCAATGAAC | 657 | 4th Exon -7th Exon |
| *BoSOC1.1.C4* | PF1:GGTCCTTTTTGAGGCTATTT | PR1: TGAGTTTTCCCCCTCACCAT | 900 | Promoter |
|  | F1: ATGGTGAGGGGGAAAACTCA | R1: CGATATTAGGAATGAGCATG | 808 | 1st Exon -1st Intron |
|  | F2: CATGCTCATTCCTAATATCG | R2: CGTTTGGAAGCTTCGAGTTG | 806 | 1st Intron-3rd Exon |
|  | F3: CAACTCGAAGCTTCCAAACG | R3: GAACAAGGTAACCCAATGAAC | 857 | 3rdExon -7th Exon |
| *BoSOC1.2.C4* | PF1:GTCCACATACAGCTAAACCA | PR1: TGAGTTTTTCCCCTCACCAT | 866 | Promoter |
|  | F1: ATGGTGAGGGGAAAAACTCA | R1: GTAGGAACATAGTAAGCAAC | 803 | 1st Exon -1st Intron |
|  | F2: GTTGCTTACTATGTTCCTAC | R2: TTCATCATGTTTGCTGCTTC | 726 | 1st Intron-3rd Exon |
|  | F3: GAAGCAGCAAACATGATGAA | R3: AAGAAGGCAACCCAATGAAC | 915 | 3rdExon -7th Exon |
| *BoLFY.C2* | PF1:CGCGTTCAGACAATGCTGAT | PR1: GTGAAACCTTCAGGATCCAT | 864 | Promoter |
|  | F1: ATGGATCCTGAAGGTTTCAC | R1: TTCTAGTGACAACTGGATAC | 765 | 1st Exon -1st Intron |
|  | F2: GTATCCAGTTGTCACTAGAA | R2: TTAGCAATGGTCTGAACCTG | 543 | 1st Intron-2nd Exon |
|  | F3: CAGGTTCAGACCATTGCTAA | R3: GGAGACACTATTACCCGCTG | 606 | 2ndExon -2nd Intron |
|  | F4: CAGCGGGTAATAGTGTCTCC | R4: CTAATTAAACCCCAAACCGC | 696 | 2nd Intron-3rd Exon |
| *BoCO.C1* | PF1: CTCTTTACATATTTCGGATG | PR1: TAATCACATTTCGGCTCCAT | 806 | Promoter |
|  | F1: ATGGAGCCGAAATGTGATTA | R1: CTCTAAAGGGTTGTCAGTAG | 835 | 1^st^ Exon -2^nd^ Exon |
|  | F2: CTACTGACAACCCTTTAGAG | R2: TCATTGATTTGTGGTTGGTG | 605 | 2^nd^ Exon - 4^th^ Exon |
| *BoCO.C3* | PF1: CCTACCACCGTGATAAACCA | PR1: ACTCTCTCGCTACTGCTCAT | 1017 | Promoter |
|  | F1: ATGAGCAGTAGCGAGAGAGT | R1: ATCTCTTTAACACCTTTGGC | 1009 | 1^st^ Exon -2^nd^ Exon |
|  | F2: GCCAAAGGTGTTAAAGAGAT | R2: CTAAGGATAAGGAGCTTCAG | 810 | 2^nd^ Exon - 4^th^ Exon |
| *BoCO.1.C4.* | PF1: GGCTCCAACCGATGACATGT | PR1: TTCTTCTTCTTTCGGATCTG | 994 | Promoter |
|  | F1: CAGATCCGAAAGAAGAAGAA | R1: CCCGCAGGTGGCGTGGCGAT | 720 | 1^st^ Exon -2^nd^ Exon |
|  | F2: ATCGCCACGCCACCTGCGGG | R2: TTATGGATGTGACTTAGCGA | 701 | 2^nd^ Exon - 4^th^ Exon |
| *BoCO.2.C4* | PF1: CTGGAGAATGTCAAACGTAC | PR1: TCGCACGGTCTCGACGCCAT | 927 | Promoter |
|  | F1: ATGGCGTCGAGACCGTGCGA | R1: TCAAAAACTCGGGACCACTC | 1023 | 1^st^ Exon -2^nd^ Exon |
| *BoCO.C5* | PF1: GCCTTCAATCGAAAAGCTTT | PR1: TCTTGACGCTCTGGAATCAT | 953 | Promoter |
|  | F1: ATGATTCCAGAGCGTCAAGA | R1: CTAGGAGTCGTTGGTTAAAG | 1067 | 1^st^ Exon -2^nd^ Exon |
| *BoCO.C9* | PF1: ACAGGTTTCCCGCCAAAATC | PR1: TGCCCCTCTGTTGTTCTCTC | 1054 | Promoter |
|  | F1: GAGAGAACAACAGAGGGGCA | R1: TCTGGTACGATGCAGTCTTG | 568 | 1^st^ Exon |
|  | F2: CAAGACTGCATCGTACCAGA | R2: GAAGGAACAATGCCATATCC | 687 | 1^st^ Exon -2^nd^ Exon |
| *BoVRN1.C1* | PF1: GCGTTATTAGGGTTACATGA | TGGAAGAAAGGGCGTGGCAT | 1020 | Promoter |
|  | F1: ATGCCACGCCCTTTCTTCCA | R1: CAACCGATAGCTCGTCCTTG | 1137 | 1^st^ Exon -2^nd^ Exon |
|  | F2: CAAGGACGAGCTATCGGTTG | R2: GGTTGGTGTTGTCTCTTCAG | 859 | 2^nd^ Exon-3^rd^ Exon |
|  | F3: CTGAAGAGACAACACCAACC | R3: TCAGACGTACTCGTTGACAC | 709 | 3^rd^ Exon- 5^th^ Exon |
| *BoVRN2.C8* | PF1: GTGTTCAGCCCTATACATAT | PR1: CGCAACAATTATGCCTACAC | 1022 | Promoter |
|  | F1: GTGTAGGCATAATTGTTGCG | R1: TCTTGATGGTTGACTTCTTC | 922 | 1^st^ Exon- 7^th^ Exon |
|  | F2: GAAGAAGTCAACCATCAAGA | R2: TGAGAATGATAGAACTGGCG | 697 | 7^th^ Exon-10^th^ Exon |
|  | F3: CGCCAGTTCTATCATTCTCA | R3: TCACTTGGCTTTGCTGTTAT | 898 | 10^th^ Exon- 14^th^ Exon |
| *BoVIN3.C3* | PF1: ATGATGAGGAATGAGGGTTC | PR1: GAGAGTTCATGAACAAGCTT | 1036 | Promoter |
|  | F1: AAGCTTGTTCATGAACTCTC | R1: GAGACACAGTAGAAGCATCC | 599 | 1^st^ Exon- |
|  | F2: GGATGCTTCTACTGTGTCTC | R2: TCACCGCTTTCTTGATATGC | 761 | 1^st^ Exon -2^nd^ Exon |
| *BoSVP.C4* | PF1: GAGGAATGTGACCTTAGGTG | PR1: TGAATCTTCTCTCTCGCCAT | 1013 | Promoter |
|  | F1: ATGGCGAGAGAGAAGATTCA | R1: CTGTAACTCAAGAGATGGCT | 1018 | 1^st^ Exon -2^nd^ Exon |
|  | F2: AGCCATCTCTTGAGTTACAG | R2: ATGCTGCCTTAGCCGCTTGT | 899 | 2^nd^ Exon-6^th^ Exon |
|  | F3: ACAAGCGGCTAAGGCAGCAT | R3: CCATACGGTAAGCTGCAAGA | 989 | 6^th^ Exon-9^th^ Exon |
| *BoSVP.C8* | PF1: CATCTCGAGGAGGGGTATTA | PR1: TGAATCTTCTCTCTCGCCAT | 584 | Promoter |
|  | F1: ATGGCGAGAGAGAAGATTCA | R1: TCTCTAACACTTCCCTCATG | 1116 | 1^st^ Exon -2^nd^ Exon |
|  | F2: CATGAGGGAAGTGTTAGAGA | R2: GTTCCTCTCCTCTCATTTGC | 634 | 2^nd^ Exon- 4^th^ Exon |
|  | F3: GCAAATGAGAGGAGAGGAAC | R3: TCTTCCGTTAACTGTGTCCC | 866 | 4^th^ Exon-7^th^ Exon |
|  | F4: GGGACACAGTTAACGGAAGA | R4: CGGTAAGCTGCACGAAGCCA | 493 | 7^th^ Exon-9Exon |
| *BoSPL.C2* | PF1: GTGGAACTCGAAACTACATG | PR1: CCGTAGCTCCAAGAATCCAT | 962 | Promoter |
|  | F1: ATGGATTCTTGGAGCTACGG | R1: CAACTTCTCTTCCCATCATC | 520 | 1^st^ Exon -2^nd^ Exon |
|  | F2: GATGATGGGAAGAGAAGTTG | R2: CTAGGGGAAATAAAGCTCAT | 551 | 2^nd^ Exon-3^rd^ Exon |
| *BoSPL.C4* | PF1: GTGAATAGGGCAAAACCAAT | PR1: TTACACACACACACACACAT | 791 | Promoter |
|  | F1: ATGTGTGTGTGTGTGTGTAA | R1: TCAGTAACCTTGAGAGCCAA | 557 | 1^st^ Exon -2^nd^ Exon |
| *BoSPL.1.C6* | PF1:GCAGTGGTGATAATAAATCC | PR1: AGATACCCTAGTCCTTGTGC | 829 | Promoter |
|  | F1: GCACAAGGACTAGGGTATCT | R1: CTATCTAATCTGTGGTCGCT | 606 | 1^st^ Exon -2^nd^ Exon |
| *BoSPL.2.C6* | PF1: CATCTGTTTGCTGCTGGTAC | PR1: CAGATACCCTTGTCCTTGTG | 1016 | Promoter |
|  | F1: CACAAGGACAAGGGTATCTG | R1: CTATCTAATCTGTGGTCGCT | 923 | 1^st^ Exon -2^nd^ Exon |
| *BoSPL.C8* | PF1: CATGTGTTACTTTGAAGAGG | PR1: TCCCATTCGTAGTCCAACAT | 1002 | Promoter |
|  | F1: ATGTTGGACTACGAATGGGA | R1: GTACGGACGGGCAAGAGGCC | 811 | 1^st^ Exon -1^st^ Intron |
|  | F2: GGCCTCTTGCCCGTCCGTAC | R2: GGAGCTAAGCTACTCCATAT | 901 | 1^st^ Intron-3^rd^ Exon |

**Table S2B.** List of previously published primers on the reported *FLC*s in *B. oleracea* used in this study.

| Gene Name | Forward (5ˊ----------------------------------3ˊ) | Reverse (5ˊ----------------------------------3ˊ) | References |
| --- | --- | --- | --- |
| *FLC1* | F: GAGGAATCAAATGTCGATAA | R: CTAATAAAGCAGTGGGAGAG C | [59] |
|  | F1: GGCTTTTGATTATGGACAAACC | R1: AACCCAACTTGGAATCAAACC | [15] |
|  | F2: GGAGTCCATCTTTCCACGTT | R2: TGATCTTGAGGTCCGGTTTC |  |
|  | F1 CCTAGACGGGTCCGAATCTGGGAC | R1 GTCCCAGATTCGGACCCGTCTAGG | [22] |
|  | F2 GTCTCTAATTGTGTTCTGTGCCC | R2 GGTTGTCTCATGTATCTAGCAAC |  |
|  | F3 GGTGTTAGAATGTATTGGCATGCCC | R3 CGGTACGGGTTCGGTTCGGATTTC |  |
|  | F4 CTTGCTCAAGGGTCCAGTGGTG | R4 GAGTGCGAAACTAAACGCTTGGG |  |
|  | F5 CATCCGTCTATTCAAACGTCCG | R5 GGCGTAGAGGTAATCCATAGAAGC |  |
|  |  | R6 GGTTGTGCATGAGGATCCATCA |  |
|  |  | R7 GCAGTGGGAGCGTTACCGGAAG |  |
|  |  | R8 GAGCTGAAGATACATGGGAGCGAG |  |
| *FLC2* | F: CCATGAGCTACTAGAACTTG | R: CTAATAAAGCAGTGGGAGAG | [59] |
|  | F3: GTAAGCTTGTGGAATCAAATTCT | ex6R: TGGCTAGCCAAACCCTGGTT |  |
|  | F1: AGGGCCTAGAGGGCATACAT | R1: TTTTGAGGCTCTCGACACAA | [15] |
|  | F2: CGAAGTATGGTTCACACCATGAGC | R2: CGGAGAGGGCAGTCTCAAGGTGGTT |  |
| *BoFLC.C2 (BoFLC4)* | F13: CGTCAGCTTTCAGTTCTCTGC | R13: CTTCGGAGCTTCTGACTGAAG | [46] |
|  | F: GTAAGCTTGTGGAATCAAATTCTGA | R: GGCTATCAACAAGCTTCAACAATAG |  |
|  | C2-1 F: CACGATTGCCATTAGAGTGGG | C2-1 R: GATTTGACCAGTATGTATGCCCTC |  |
| *FLC3* | F: GTGGAATCAAATGTCGGTGG | R: CTAATAAAGCAGTGGGAGAG | [59] |
|  | 2 F1: AGCAGAAACGGGAACCTACA | 2 R1: CATTGCCATTTACTGCATCG | [15] |
|  | 2 F2: CCTCCTCCGGAAAGCTCTACAGC | 2 R2: TGTCCACGCTTACACCAACGAC |  |
|  | F1: GGTACACGTGGCTGTCTTCTCGTC | R1: CGGGTACCCGAAATATTTCGGTTC | [22] |
|  | F2: GAGGTATTGCATTGTTGGTCCACC | R2: GTCAATAGCTGGACAATGTCGTAC |  |
|  | F3: GTGCCGGTGTTCATTCAAATTTGG | R3: CCAGGGCTTTAAGATCATCAGC |  |
|  |  | R4: GACTGAAGATCCTGTCCACGGAG |  |
|  |  | R5: CAAGAAGTGCTTATCGGCTTTTGC |  |
|  |  | R6: CTCCATATTATCAGCTTCGGCTCCC |  |
|  |  | R7: GGAGTACACACAATCTCTCAGCC |  |
| *FLC4* | F1: CTCCTCTTCAGCCTGGTCAAGGTC | R1: CTAACAAAAACGCCCTTCTCGGC | [22] |
|  | F2: GACAGGATCTTCAGTCAGAAGCTCC | R2: GGGCATCTCCGTCCCAACTCCAT |  |
|  | F3: CGAATGTATGCCACATTGTGCAGC | R3: GGAGCTTCTGACTGAAGATCCTGTC |  |
|  | 1 F1: GAAATATGGAAAGCGGGTGA | 1 R1: AGAGGTGATGCGCCTAGAAA | [15] |
|  | 1 F2: GGGATTGCGCAAAATTCTAA | 1 R2: GCGTGGACGGCTAGTGTATT |  |
| *FLC5* | c F: AGTGTGGAAGGGATGTGAAAG | c R: TGAGGTTACAGACGTCTAAC | [59] |
|  | extra-band F: TGGAATCAATTGTCGATGTA | extra-band R: CTAATAAAGCAGTGGGAGAG |  |
|  | 33F: AGTGTGGAAGGGATGTGAAAG | 33R: TGAGGTTACAGACGTCTAAC |  |
|  | F1: CCATTCGAGTTCGGATATTG | R1: CGATGCGTTTTAACGACAAG | [15] |
|  | F2: TGATGAAGGAAGACCCTTGG | R2: TTCTCTATCATGGCGGTTCC |  |
|  | F3: ATCGGCAATCGTTGTGTGTA | R3: CCCAAGACAAAAGACCCAAG |  |
|  | F1: GCGGTGCACGTGGCTGTCTTGTCG | R1: CGGAGGAGAAGCTGTAGAGCTTG | [22] |
|  | F2: GAGAGATCTCAGAATATACTCTCG | R2: GATTCGCCGGGTAAATCTAAGTGTC |  |
|  | F3: CTGAATGCTAGGTTCAGCCTTGG | R3: CGCGAAGAGACAGCCAACGGTATC |  |
|  | F4: GGTAGATTCCAGTGGTGTCTTC | R4: GGTATCAGAGGGTTCTAGCGATCC |  |
|  | F5: ATACACTGGTCCTTTACCGCCTC | R5: GACTAATGGAACTCGGCACTAAC |  |
|  | F6: GCTTTCCTAGCTAGTTCAGCCAGG | R6: CAACTGATGCACATTACGTGCTGC |  |
|  |  | R7: CAGGGCGTGTGTTGCTGCACTTCC |  |
|  |  | R8: CCAGGGCATTGAGATCATCAGC |  |
|  |  | R9: CATTTGTAATGAAAGGAGGAGAGC |  |
|  |  | R10: CCTGGCTGAACTAGCTAGGAAAGC |  |
|  |  | R11: GCAGCGGAAAGCAAAACCTACATC |  |
