# Supplementary material for: Molecular markers based on sequence variation in BoFLC1.C9 for characterizing early- and late-flowering cabbage genotypes
Source: BMC Genet. 2019 Apr 27;20:42. doi: 10.1186/s12863-019-0740-1 (PMC6487051; doi:10.1186/s12863-019-0740-1)
Supplement: Supplementary file 2 — Figure S1. PCR amplicons of BoFLC1.C9, BoFLC3.C3 and BoFLC4.C3 in late-flowering line BN3848 (P1) and early-flowering line BN623 (P2). PCR products with respective primers from start to stop codons of the genes (Additional file 1: Table S1A) were run on a 1.5% agarose gel and their corresponding amplicon sizes are mentioned. M is a 100-bp size marker. Figure S2. Sequence alignments of the BoFLC1.C9 gene cloned from early- and late-flowering lines. Variation of a 67-bp insertion in the early-flowering line BN623 is highlighted in red color. Figure S3. PCR-amplicons of 141 F2 segregating population with F7R7 primers of Indel marker of BoFLC1.C9 gene. P1 = Late-flowering parent (BN3848), P2 = Early-flowering parent (BN623), F1 = (BN3848 × BN623); black and red colored numbers of the F2 individual are matched and mismatched lines, respectively as early- and late-flowering lines. M = 100 bp DNA marker. Figure S4. Regression and correlation coefficient between marker dosage and phenotypes as days to flowering after sowing (DAS) explained by the F7R7 marker in 141 F2 individual. ** indicates p < 0.01. (PPT 2295 kb) [file 12863_2019_740_MOESM2_ESM.ppt]

## Slide 1
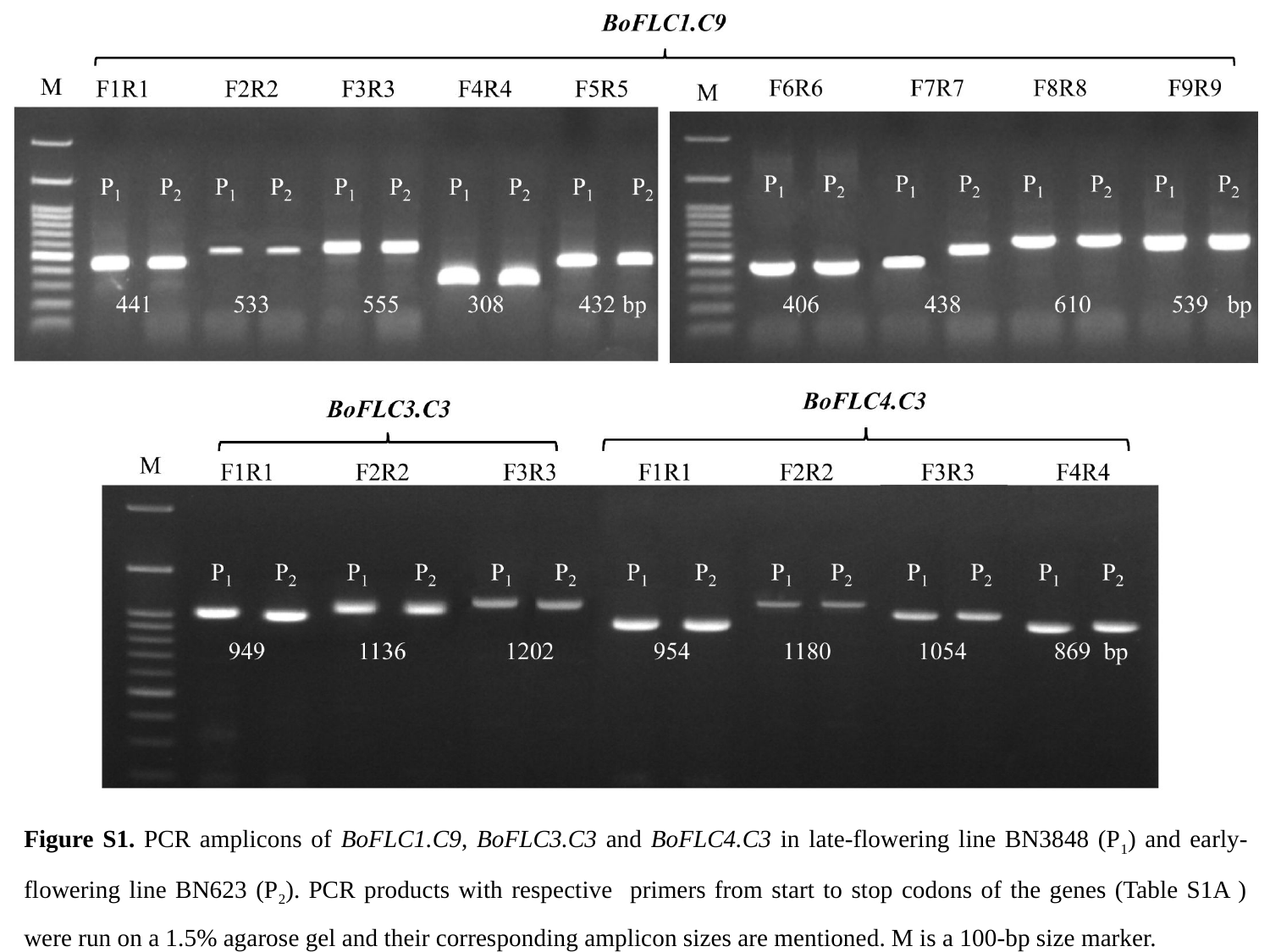

Figure S1. PCR amplicons of BoFLC1.C9, BoFLC3.C3 and BoFLC4.C3 in late-flowering line BN3848 (P1) and early-flowering line BN623 (P2). PCR products with respective primers from start to stop codons of the genes (Table S1A ) were run on a 1.5% agarose gel and their corresponding amplicon sizes are mentioned. M is a 100-bp size marker.

## Slide 2
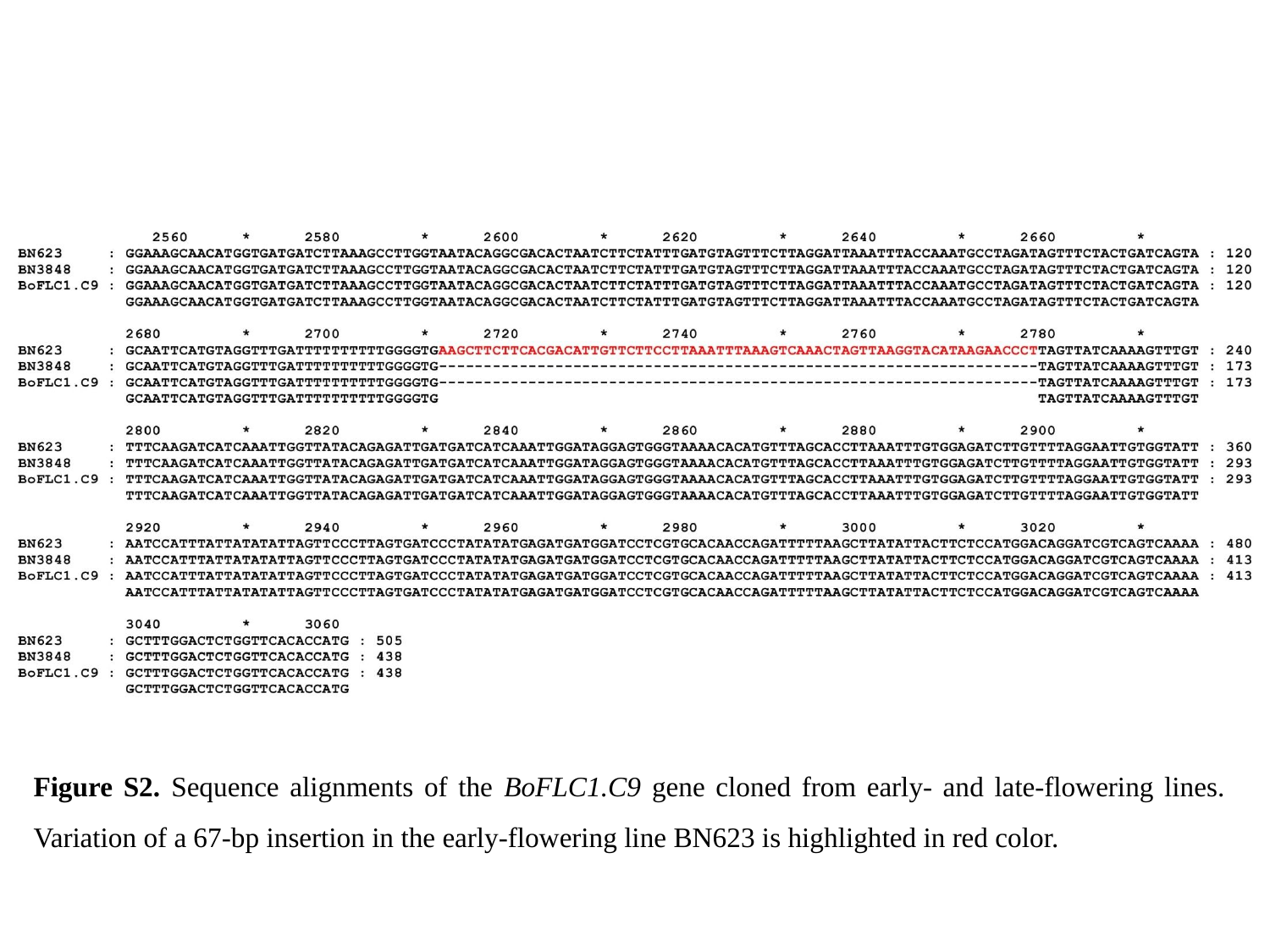

Figure S2. Sequence alignments of the BoFLC1.C9 gene cloned from early- and late-flowering lines. Variation of a 67-bp insertion in the early-flowering line BN623 is highlighted in red color.

## Slide 3
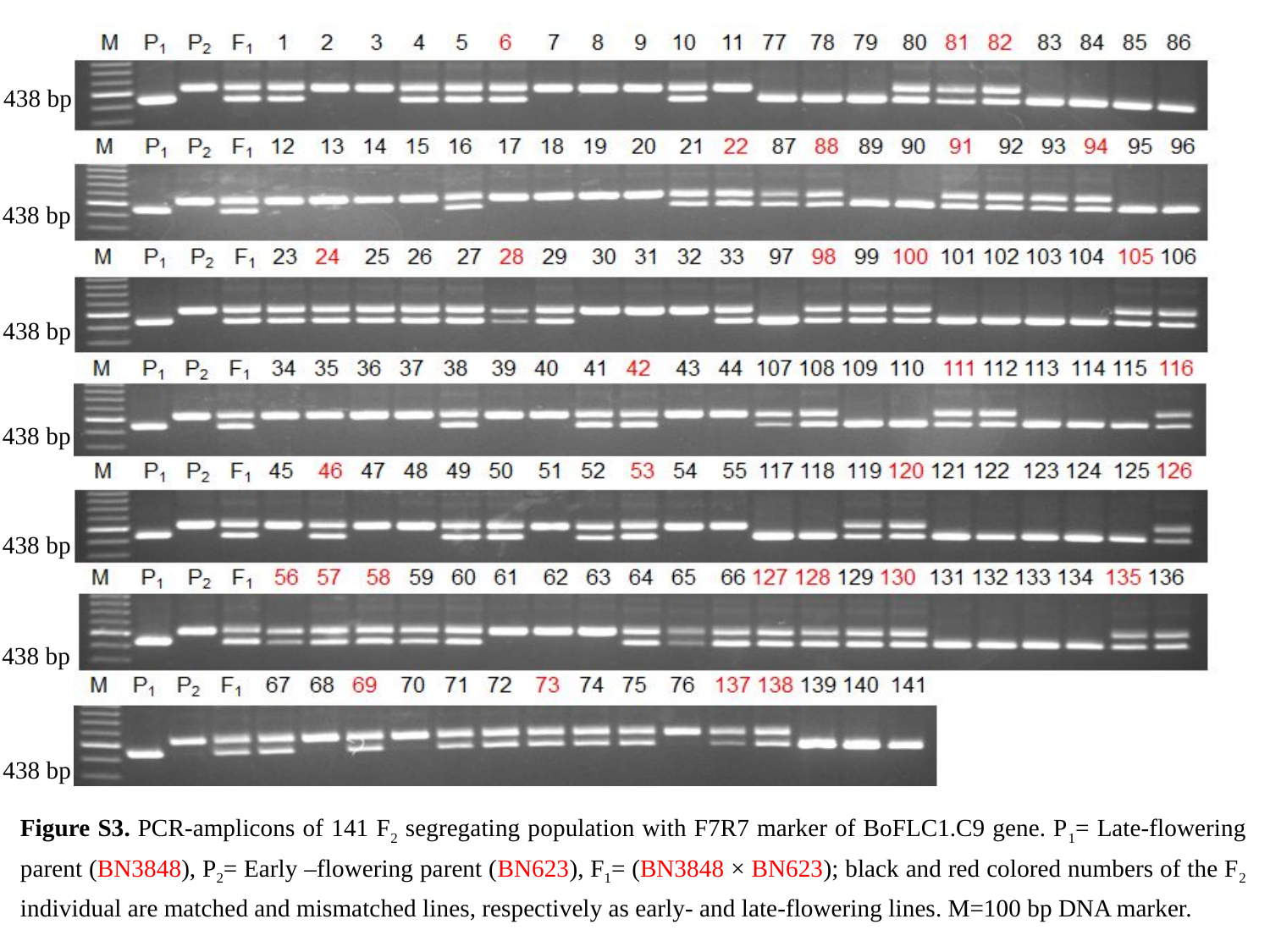

438 bp
438 bp
438 bp
438 bp
438 bp
438 bp
438 bp
Figure S3. PCR-amplicons of 141 F2 segregating population with F7R7 marker of BoFLC1.C9 gene. P1= Late-flowering parent (BN3848), P2= Early –flowering parent (BN623), F1= (BN3848 × BN623); black and red colored numbers of the F2 individual are matched and mismatched lines, respectively as early- and late-flowering lines. M=100 bp DNA marker.

## Slide 4
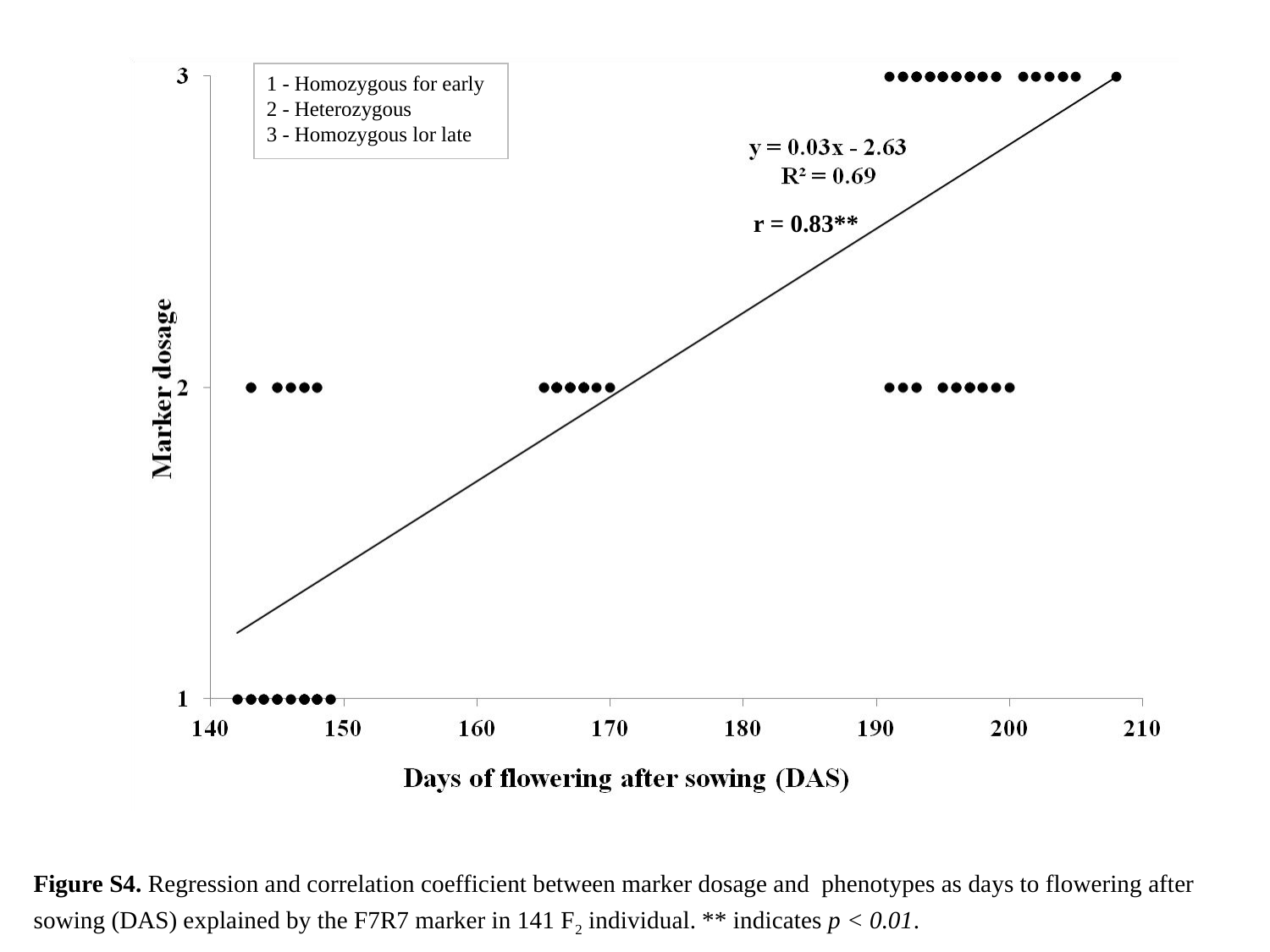

1 - Homozygous for early
2 - Heterozygous
3 - Homozygous lor late
r = 0.83**
Figure S4. Regression and correlation coefficient between marker dosage and phenotypes as days to flowering after sowing (DAS) explained by the F7R7 marker in 141 F2 individual. ** indicates p < 0.01.
