# Supplementary material for: Molecular markers based on sequence variation in BoFLC1.C9 for characterizing early- and late-flowering cabbage genotypes
Source: BMC Genet. 2019 Apr 27;20:42. doi: 10.1186/s12863-019-0740-1 (PMC6487051; doi:10.1186/s12863-019-0740-1)
Supplement: Supplementary file 3 — Sequence alignment of the total sequences of the gene BoFLC3.C3 and BoFLC4.C3 cloned from early-flowering line (BN623) and late-flowering line (BN3848) aligned with reference sequence of BoFLC3.C3 and BoFLC4.C3 genes, respectively. Red highlighted letter indicate SNPs variations in the early-flowering line. Forward and reverse arrows indicate forward and reverse primer sets used for cloning and sequencing. (DOC 747 kb) [file 12863_2019_740_MOESM3_ESM.doc]

**Additional file 3.** Sequence alignment of the total sequences of the gene *BoFLC3.C3* and *BoFLC4.C3* cloned from early-flowering line (BN623) and late-flowering line (BN3848) aligned with reference sequence of *BoFLC3.C3* and *BoFLC4.C3* genes, respectively. Red highlighted letter indicate SNPs variations in the early-flowering line. Forward and reverse arrows indicate forward and reverse primer sets used for cloning and sequencing.

1. For *BoFLC3.C3* gene

1. For *BoFLC4.C3* gene
